# Resilience amongst Ontario registered practical nurses in long‐term care homes during COVID‐19: A grounded theory study

**DOI:** 10.1111/jan.15453

**Published:** 2022-10-11

**Authors:** Denise M. Connelly, Anna Garnett, Nancy Snobelen, Nicole Guitar, Cecilia Flores‐Sandoval, Samir Sinha, Jen Calver, Diana Pearson, Tracy Smith‐Carrier

**Affiliations:** ^1^ School of Physical Therapy Western University London Ontario Canada; ^2^ Arthur Labatt Family School of Nursing Western University London Ontario Canada; ^3^ Registered Practical Nurses Association of Ontario (WeRPN) Mississauga Ontario Canada; ^4^ Health and Rehabilitation Sciences Western University London Ontario Canada; ^5^ University of Toronto Toronto Ontario Canada; ^6^ Lambton College Sarnia Ontario Canada; ^7^ School of Humanitarian Studies Royal Roads University Victoria British Columbia Canada

**Keywords:** adult nursing, grounded theory, long‐term care, practice development, qualitative approaches

## Abstract

**Aims:**

This study aimed to understand how the personal and professional resilience of Registered Practical Nurses working in long‐term care (LTC) homes in Ontario were impacted during the Coronavirus 2019 pandemic.

**Background:**

Registered Practical Nurses are primary regulated healthcare providers that have worked in Ontario LTC homes during the COVID‐19 pandemic. As frontline workers, they have experienced increased stress secondary to lockdowns, changing Ministry of Health recommendations, social isolation and limited resources. LTC homes experienced almost a third of all COVID‐19‐related deaths in Ontario. Understanding registered practical nurses' (RPNs) resilience in this context is vital in developing the programs and supports necessary to help nurses become and stay resilient in LTC and across a range of settings.

**Methods:**

Purposive sampling was used to recruit 40 Registered Practical Nurses working in LTC homes across Ontario for interviews. Charmaz's Grounded theory guided in‐depth one‐on‐one interviews and analyses completed between April to September 2021.

**Results:**

Registered Practical Nurse participants represented 15 (37.5%) private, and 25 (62.5%) public LTC homes across Ontario Local Health Integration Networks. Findings informed two distinct perspectives on resilience, one where nurses were able to maintain resilience and another where they were not. Sustaining and fraying resilience, presented as bimodal processes, was observed in four themes: ‘Dynamic Role of the Nurse’, ‘Preserving Self’, ‘Banding Together’ and ‘Sense of Leadership Support’.

**Conclusion:**

Resilience was largely drawn from themselves as individuals. Resources to support self‐care and work‐life balance are needed. Additionally, workplace supports to build capacity for team‐based care practices, collegial support in problem‐solving and opportunities for ‘connecting’ with LTC nursing colleagues would be beneficial. Our findings suggest a role for professional development resources in the workplace that could help rebuild this workforce and support RPNs in providing quality care for older adults living in LTC.

**Patient or Public Contribution:**

Our research team included two members of the Registered Practical Nurses Association of Ontario, and these team members contributed to the discussion and design of the study methodology, recruitment, analysis and interpretation. Further, RPNs working in long‐term care during the COVID‐19 pandemic were the participants in this study and, therefore, contributed to the data. They did not contribute to data analysis or interpretation.

**Impact**

**What problem did the study address?**

- Resilience‐based educational strategies are used for developing skills in nurses to respond positively to adversity.
- Throughout the COVID‐19 pandemic, RPNs working in Ontario long‐term care (LTC) homes experienced prolonged lockdowns, challenging working conditions and inadequate resource allocations that have compounded the strain on this historically neglected healthcare workforce.
- Previous research findings from an integrative review of resilience in nurses concluded that understanding resilience can assist in providing support and developing programs to help nurses become and stay resilient.

**What were the main findings?**

- This is the first study exploring the nature of individual resilience through the voices of RPNs working in Ontario's LTC sector during the COVID‐19 pandemic.
- Fueling or draining individual resilience, presented as bimodal processes, were discovered in four themes: ‘Sense of Leadership Support’, ‘Banding Together’, ‘Dynamic Role of the Nurse’ and ‘Preserving Self’.

**Where and on whom will the research have an impact?**

- The insights into the nature of individual resilience from nurses themselves may inform nurses and employer organizations to support and retain novice and expert nurses in the LTC sector.
- The findings suggest there is a role for professional development and workplace solutions for supporting the recruitment and retention of the workforce so they can care for people living in LTC.
- Resilience is important for the retention and recruitment of nurses in LTC homes and future studies need to examine this construct in relation to objectively measured staff and resident outcomes.

## 1 INTRODUCTION

Registered practical nurses (RPNs), the largest regulated health professional workforce in Ontario long‐term care (LTC) homes, have been at the frontline of the Coronavirus Disease 2019 (COVID‐19) pandemic (Odom‐Forren, 2020). The workforce of the LTC sector has been significantly impacted by the ongoing COVID‐19 pandemic, with 79% of all COVID‐19‐related deaths in Canada occurring in LTC homes (Rothan & Byrareddy, 2020). Only 54% of healthcare providers working in LTC have agreed that following provincial COVID‐19 recommendations (e.g. use of personal protective equipment [PPE], physical distancing of 6 m) was a feasible strategy for managing the pandemic (Siu et al., 2020), highlighting a lack of preparedness in the processes of the LTC sector in the face of crisis. Further, this sector experienced challenges even before the pandemic, such as chronic staffing shortages, low staffing levels, heavy workloads, punitive measures for staff experiencing illness, environmental deficiencies, lack of infection control processes and high levels of self‐reported staff burnout (McGilton et al., 2020; White et al., 2021). Researchers outside of Ontario have also suggested that interventions to improve LTC staff resilience are critical to revitalizing this workforce (Williams, Hadjistavropoulos, et al., 2016; Williams, Lum, et al., 2016). The aim of this study was to understand how the personal and professional resilience of RPNs working in LTC homes in Ontario were impacted during the coronavirus disease‐2019 (COVID‐19) pandemic.

### 1.1 Background

The workforce in Ontario LTC homes has been particularly vulnerable to COVID‐19. In 2020 in Canada, the largest proportion of deaths from COVID‐19 occurred in Ontario LTC homes (i.e. 79%; Rothan & Byrareddy, 2020). From the onset of COVID‐19 and throughout 2020 in Canada, Ontario LTC homes were epicentres in the fight against infection transmission. The statistics fail to capture the disturbing social, emotional, psychological and physical impacts the pandemic had on healthcare workers, residents and families. Going forward, it will be important to understand how RPNs experienced a crisis like COVID‐19 to make changes necessary to support individual, organizational, policy and social factors that have shaped their experiences. In Ontario LTC homes, a variety of nurses work, including RPNs and registered nurses; however, RPNs have been at the forefront of the COVID‐19 pandemic as the largest regulated health professional workforce in Ontario LTC homes (Odom‐Forren, 2020) and are therefore the focus of this study.

In 2019, the Ontario Health Coalition reported a failure to plan and resource the LTC system, despite the predictability of aged population health trends, including the increasing prevalence of dementia and greater complexity of chronic healthcare needs. Currently, as evidence of and response to, the historically underfunded staffing of LTC facilities and the lack of updates in the infrastructure of these facilities, two new national standards for LTC are in development: HSO 21001:2020—Long‐Term Care Services and HSO Infection Prevention and Control Standards (https://longtermcarestandards.ca/about‐standard). During COVID‐19, RPNs, residents and other members of the workforce (e.g. Personal Support Workers [PSWs]), have been negatively impacted by the chronic neglect of the sector, demonstrated by heavy workloads, rushed work environments and inadequate collaborative team care (e.g. established protocols and policies; White et al. 2021). Throughout COVID‐19, social isolation has emerged as a representative theme of RPN and resident experience in LTC (Chu et al., 2020) resulting from little time for humanistic nurse‐resident relationships and a failure to maintain the emotional and psychological needs of residents and nurses (Williams, Hadjistavropoulos, et al., 2016; Williams, Lum, et al., 2016). In Japan, researchers have found that 10% of healthcare workers (i.e. doctors, nurses, other interdisciplinary staff and office workers) developed moderate‐to‐severe anxiety disorders, and roughly 28% developed depression during COVID‐19 (Awano et al., 2020).

The construct of resilience has been identified by the Registered Practical Nurses Association of Ontario (WeRPN) as being central to nurses. Informed by Ungar's (2018) ecological model of resiliency, this construct is understood ‘as a sequence of systemic interdependent interactions through which actors (whether persons, organisms, or ecosystems) secure the resources required for sustainability in stressed environments’ (Ungar, 2018, p. 33). In this study, resilience in RPNs is viewed holistically, as integrating personal, professional and organizational ‘recovery responses’ to challenging experiences. Recovery responses may include but are not limited to, self‐care activities, targeted education, timely communication from leadership, acknowledgement of demands on workers, opportunities for open discussion and reflection with colleagues and professional development. Ungar (2018) outlines personal resilience as a process by which people ‘bounce back’ from adversity, frustration and misfortune using the psychological and biological strengths humans employ to cope with challenges and threats (Ungar, 2018). Professional resilience addresses the capacity of individuals to thrive in demanding workplace situations, exemplified by a willingness to act in responding to difficult situations (Ungar, 2018).

Previous research, addressing how the conceptualization of resilience in nursing influences interventions aimed at increasing resilience, reported that resilience‐based educational strategies are used for developing skills in nurses to respond positively to adversity (Stacey & Cook, 2019). However, understanding the nature of resilience in RPNs working in the LTC sector, beyond experiencing the death of residents, is lacking. Rochat et al. (2017) identified risk and protective factors influencing career resilience: the negative effects of limited resources (e.g. material, human capital, social support), and alternatively, strong social support and breadth of skills (i.e. protective factors). Recently, Hite and McDonald (2020) have suggested that both individual and contextual factors influence nursing career resilience.

Ungar (2018) suggests that even when a person's individual adaptive resilience processes are present, social and physical ecologies or environmental antecedents, are major players influencing positive outcomes when individuals and organizations are under stress. Conceptualizing resilience in RPNs holistically draws together individual, professional and organizational factors to inform a broad view of the process of how RPNs working in LTC homes in Ontario build and sustain personal and professional resiliency during a crisis such as COVID‐19. This research seeks to contribute knowledge necessary to develop resources to support RPNs, so that they are less vulnerable as the workforce in this sector, in the event of a future pandemic or health crisis.

## 2 THE STUDY

### 2.1 Aim

This study aimed to understand how RPNs working in LTC homes in Ontario may have built and sustained personal and professional resilience during the COVID‐19 global pandemic.

### 2.2 Design

Constructivist grounded theory methodology (Charmaz, 2006) was used in this study to interpret the process of how RPNs maintain resilience during a crisis. Consistent with a constructivist lens, the knowledge generated in this research was shaped through an interactive dialogue between the researcher(s) and the research participants and the actions and processes are specific to context and experiences (Charmaz, 2000, 2006). The findings of this study could, therefore, have analytical generalizability; that is, the results could lead to conceptual insights that provide new ways of considering issues in other settings (Charmaz, 2006).

Constructivist grounded theory is particularly appropriate to use when a study seeks to understand and conceptually map out a process. Such a process can be defined as ‘unfolding temporal sequences that may have identifiable markers with clear beginnings and endings and benchmarks in between. The temporal sequences are linked in a process and lead to change’ (Charmaz, 2006, p. 10). This study explored the process of building and maintaining resilience amongst RPNs working in LTC during COVID‐19. Inductive guidelines for data collection and analysis were employed to build a theoretical framework that would help interpret the data gathered (Charmaz, 2000). Methods of participant recruitment and selection, data collection and analysis are detailed below. The Consolidated Criteria for Reporting Qualitative Studies (COREQ; Tong et al., 2007) was used throughout the study design, data collection and analysis and in the writing of this manuscript.

### 2.3 Sample of participants

RPNs working, or who had worked, in LTC homes in Ontario since January 2020 during the COVID‐19 pandemic were eligible; nursing students, other categories of nurses (e.g. Registered Nurses [RNs]), or other healthcare professionals or staff were not. Eligible RPNs were invited to complete an online survey that aimed to describe the personal and professional resilience of RPNs working in LTC during the COVID‐19 pandemic. Invitations to anonymously participate in the online survey were sent to registered RPNs by the WeRPN. Results of the survey are reported elsewhere by this study's authors. A letter explaining the purpose of the interview was provided at the end of the survey, and potential participants were invited to provide their contact information if they were willing to be interviewed by a research team member. Participants provided oral consent at the beginning of the interview, prior to the audio recording. All participant's data were deidentified and participants were able to withdraw consent for participation at any time. We used maximum variation and purposive sampling (Patton, 2002) to gain a broad representative sample of participants (e.g. age, gender, infection rate, geographic location, years employed as an RPN in LTC). Charmaz supports the notion of ‘sampling adequacy’ and redundancy in categories whereby sampling continues until no new knowledge is constructed or interpreted from the data collected.

### 2.4 Data collection

The series of one‐on‐one, in‐depth, semi‐structured interviews were conducted between April and September of 2021. A research team member [CFS], female PhD Candidate and postdoctoral associate working on the research project completed the interviews using a secure, institutional Zoom platform. The interviewer did not have a relationship with participants prior to study commencement nor were any characteristics about her described to the participants (e.g. biases, assumptions). Potential participants were informed of the study's purpose through the letter of information. No person other than the participant and researcher was present during the interviews.

With the permission of participants, each interview was digitally video‐ and audio‐recorded using Zoom software for transcription purposes. Participants chose when the interviews were held, including the time of day and which day of the week. A semi‐structured interview guide was pilot tested through the research team and asked questions such as: (personal resilience) *How do you ‘come back’ after a personally challenging event? How did COVID‐19 influence your ability to ‘bounce back’*; and (professional resilience) *Tell me about your biggest challenge in your current role during COVID‐19? How does your professional training as a nurse equip you to manage patient care in long‐term care? Do you feel that your colleagues in long‐term care supported you during the COVID‐19 pandemic; if so, how?* Probing questions were added, as necessary, to gain more information (e.g. ‘what do you mean when you say X?’). Reflective notes were made after each interview to capture participants' nonverbal communication and to provide an opportunity for the research team to debrief. Interview data was analysed throughout data collection. No interviews were repeated.

### 2.5 Ethical considerations

This study was approved by Western University's Institutional Review Board (Study ID #118628). The RPNs who gave their consent to participate in the study were informed that they could withdraw from the study at any time or decline to respond to any question they preferred not to answer. Confidentiality was maintained by removing all identifying features of participants from the interview transcripts. All data was stored in a secure institutional one drive that was only accessible to study investigators. As per University of Western Ontario guidelines, data will be stored for a period of 7 years prior to being deleted. For the purposes of this manuscript, numbers have been used to identify participants instead of names.

### 2.6 Data analysis

All interviews were transcribed verbatim. The process began by reading through interview transcripts several times and becoming familiar with the data to comprehend its essential features. The reflexive notes and transcribed interviews were analysed using the constructivist grounded theory approach outlined by Charmaz (2006). Strategies included a two‐step data coding process, comparative methods, reflective note writing to build conceptual analyses and the integration of a theoretical framework (Charmaz, 2000). Data were analysed throughout data collection using a social constructionist grounded theory approach to address the ‘how’ and ‘why’ questions underlying RPN stories of resilience.

Data were coded in a line‐by‐line fashion by two authors using NVIVO Version 2 (2006; QSR International Pty Ltd), a qualitative research software package. This software was used as an organizational, not analytical, tool. The analysis then progressed from line‐by‐line to focused coding, allowing codes to be brought together across observations and participants to create units of meaning (i.e. categories; Charmaz, 2004). A constant comparative method of analysis was used, comparing data generated in research participants' interviews, between participants, comparing experiences with other experiences and comparing the data generated by interviews in each category and between categories in conjunction with the interviewer's reflective notes (Charmaz, 2006). Analysis of de‐identified transcribed interviews was ongoing as interviews were completed. Minor wording revisions were vetted by members of the research team to improve the clarity of the questions. This multi‐stage coding and developing of categories resulted in the creation of a conceptual model of the process of how RPNs' personal and professional resiliency is either fueled or drained during a crisis such as COVID‐19. Emerging ideas, concepts and the theoretical model were continually discussed with members of the research team to facilitate researcher reflexivity and enhance theoretical sensitivity. Transcripts were not returned to participants for comment.

### 2.7 Rigour

Quality criteria proposed by Charmaz (2006, 2014), originality, resonance and usefulness, were observed. Conceptualizing the resilience of nurses as a product of individual and professional factors may be viewed as offering a fresh way (originality; Charmaz 2017) to consider the recognized problem of ‘burnout’ in the nursing workforce. Resonance demonstrates that the researchers have constructed concepts that not only represent their research participants' experience but also provide insight to others. Authentic citations were used to illuminate the constructed concepts to represent the research participants' experience (resonance; Charmaz 2017). The study findings presented to the WeRPN Board of Directors, which includes RPNs, were confirmed to be applicable to their own experiences, and what members had relayed to the professional association. Findings of the study were collated into a Consultation Workbook in response to a call for reform of the Health Standards Organization National Long‐Term Care Services Standard and contributed to a Report (HSO, 2022) with the aim to inform policy and practice applications (usefulness; Charmaz 2017). Transcripts were not returned to participants for comment and/or correction.

## 3 FINDINGS

The participant sample (*n* = 40) included 38 women (95%) and 2 men (0.05%), representing recruitment from 15 private (37.5%) and 25 public (62.5%) LTC homes in Ontario across all Ontario Local Health Integration Networks (LHINs) and four of the five Ontario Health Transitional Regions (see Table 1). There was no attrition. The average duration of an interview was 41.41 min (SD = 9.23 min, minimum time = 18.29 min, maximum time = 54.36 min). Participants' ages were not recorded to maintain anonymity. A total of 31 participants (77.5%) reported that their LTC home had been in a COVID‐19 outbreak at some point since the pandemic began, whilst 9 participants (22.5%) reported that their workplace had never been declared to be in an outbreak.

**TABLE 1:** Participant distribution across Ontario health interim and transitional regions.

Region	Participant, *N* (%)
West	18 (45)
Central	7 (17.5)
Toronto	0 (0)
East	10 (25)
North	5 (12.5)

**FIGURE 1:**
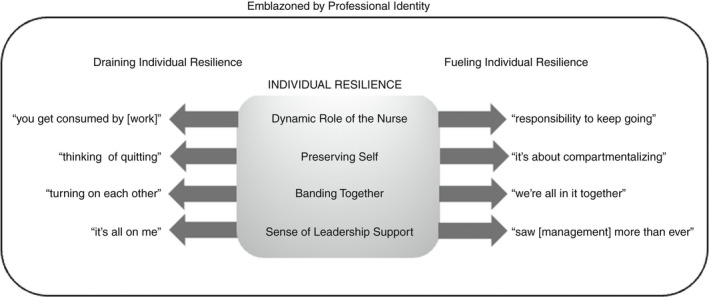
The process of maintaining resilience amongst RPNs working in LTC during COVID‐19 required navigation through various contextual elements fueled by a desire to reconnect with their sense of professional identity as a nurse. Four themes are illustrated with examples of how that theme either leads to a drain on, or fuel for, individual resilience.

Experiences storied by RPNs from working in LTC during the COVID‐19 pandemic highlighted how RPNs described resilience (i.e. their capacity to adapt in the face of challenge). After 40 interviews, data saturation suggested that four themes had emerged: ‘Dynamic Role of the Nurse’, ‘Preserving Self’, ‘Banding Together’ and ‘Sense of Leadership Support’ (see Figure 1). These themes were regarded by RPN participants in distinct ways, either as a fuel or drain on their individual resilience. The process of maintaining individual resilience by RPNs was, at its essence, ‘Emblazoned by Professional Identity’ and fundamentally central to how they viewed themselves as nurses (i.e. the process of maintaining individual resilience by RPNs was governed by their professional identity as a nurse). This process is conceptualized as continually recurring whenever resilience was required from RPNs working in LTC and represents a process occurring in and over periods of time when RPNs were required to call on their capacity to adapt to challenge(s).

### 3.1 Dynamic Role of the Nurse: ‘Responsibility to keep going’ vs. ‘Thinking of quitting’

In the first theme, ‘Dynamic Role of the Nurse’, RPNs talked about their function as a nurse, coupled with either a sense of responsibility to ‘keep going’ [T27; T34; T68] and push through ‘no matter what’ [T68]; or thoughts about quitting because of a perceived lack of support and burnout. Importantly, several participants underscored their sense of responsibility to the residents and upholding a high standard of nursing care. This inherent desire to serve as a sense of support to the residents in LTC over the difficult first year of the pandemic was further stressed as family and/or care partners were prohibited from entering LTC homes. The way RPNs perceived their role as a nurse was foundational to their professional identity and fueled their responsibility to keep going:

There's a professional standard… that never waivers, no matter what's going on, if there's a pandemic or not. I know what my responsibilities are, and I know what I have to do. I know what my professional obligation is [T68].

RPNs talked about resilience as the ability to ‘just keeping going no matter what’ [T34]. Ultimately, this idea is deeply connected to their professional identity as a nurse and the perception that they were obligated to continue giving of themselves, without receiving additional support. Moreover, they felt strongly that ‘resilience is a skill nurses have’ [T44], and that it is central to the profession:

I just keep going. I mean, resilience is the ability to bounce back from any situation. And I have always been able to. I don't know if I'm strong willed, or stubborn, or what. But you just keep going [T27].

The idea that nurses need to be able to ‘just keep going’ [T27] was found to either fuel or drain an individual's resilience. RPNs were apt to think of themselves as wearing ‘iron suits of armour’ [T64] in the LTC culture:

Resilience … means just keeping going with what you are doing, no matter what challenges you're facing [T34].

The notion of keep going ‘no matter what’ [T34] offered RPNs a ‘badge of honour’ in giving so much of themselves for the profession. One RPN described this type of sacrifice, to keep going ‘no matter what’ [T34], as ‘part of the job description’ [T68].

As a nurse, you have to keep going … it's kind of almost just part of the job description [T68].

This interpretation of their role as having no limits frames a fundamental component of their professional identity. However, when RPNs believed they were unable to keep going, they reported having to constantly compromise, putting themselves at risk and feeling as though they were at a crossroads about whether they had the capacity to continue their work. This was manifested as a drain on their resilience and compromised their perception of themselves as nurses:

I feel like I'm constantly compromising, and it's not a good feeling [T65].

Whilst compromise, or accepting lower than optimal standards, in the face of an unprecedented global pandemic may be an appropriate response for self‐preservation, many RPNs struggled to accept that they might not have the constant capacity to adapt:

I'll get pulled here and pulled there. And that just gets very frustrating when my work isn't getting done. And then, you know, I'm struggling in my work … I'm not giving 100% [T33].

The uncompromising nature of their role was deemed to be a drain on RPNs' sense of individual resilience. As such, some voiced concerns about quitting, changing careers, or changing work locations:

I'm just at a crossroads and I'm probably going to quit my job so, and I don't feel very resilient [T35].

This sentiment affirms the belief that RPNs must be constantly resilient in their role and discount the adverse impacts associated with prolonged periods of chronic stress, workload increases, and staffing shortages as contributors to their purported ‘crossroads’ [T35]. Some RPNs were able to identify the factors in the job external to themselves that contributed to their drained resilience:

I start weighing things like how much [money] I'm making as a nurse, versus let's say how much I could make at another job, and not be as stressed out, so it's like money versus mental health and well‐being [T43].

The fact that some RPNs believed that they could financially succeed just as well in a different profession, but that they chose to remain working in LTC, speaks to their profound attachment to their professional identity. One RPN described the conflict in her nursing role whilst thinking about quitting; she resolved that she truly loved her work. She described conflict with the previously expressed idea that ‘resilience is a skill that nurses have’ [T44] and acknowledged how she felt unable to ‘bounce back’ [T88]. This tension caused her to question if she should continue to be a nurse:

[The pandemic] made me really consider for a brief period of time, ‘is nursing right for me, should I choose a different career?’ And I really can't see myself right now doing anything else ‐ this is what I love. But there are definitely times when I thought maybe this isn't for me … maybe I shouldn't be a nurse because I can't bounce back from going through that scared, treacherous experience [T88].

The belief that nurses must have resilience and be able to rapidly bounce back in the face of adversity was a drain for some RPNs, and conversely, fuel for others. This divergence was dependent on their perception of their ‘responsibility [to keep going]’ [T68]. Other participants described the longstanding impacts that negative experiences had on their well‐being. For instance, some RPNs expressed that they continue to relive traumatic experiences that happened to them over the course of the pandemic. They talked about readjusting their expectations of themselves to keep going:

There's [sic] days that I have flashbacks and I just start crying. Like I came out of work last week, two weeks ago and I just cried for the rest of the day, but it teaches you that, no matter what happens, you do have the power to keep going. You might not finish everything you wanted to in that shift but that's when you have to accept that [T85].

Acceptance by the RPNs that they could ‘compromise’ [T65] whilst maintaining their ‘professional obligation[s]’ [T68] was important to augment their individual resilience, rather than deplete it. This was also reflected by RPNs describing that with the pandemic they had begun to prioritize what work was essential during a shift instead of skipping their breaks, never taking days off and constantly working overtime:

I have to sort of be better at … triaging … what needs to be done [T65].

RPNs talked about the importance of ‘saying no’ to preserve their resilience and avoid ‘stretch(ing)’ themselves too thin:

Nurses are pretty bad at saying no to a lot of things … I know if I pushed myself to try and fit that [extra task] in that it's not worth the personal toll it's going to take on me to stretch myself [T14].

Understanding that their sense of professional identity can be maintained even when they ‘say[ing] no’ [T14], despite a sense of ‘obligation [to complete a task]’ [T68], fueled RPNs to keep going. One recurrent realization from the RPNs who found their ‘dynamic role as a nurse’ as a source of fuel for their individual resilience was that taking breaks was essential:

One thing that I actually started doing, which I never did before, was taking my afternoon break. I always would, before COVID, would rush through my afternoon without taking any breaks. So now I made sure that I had my regular breaks to be off the unit [T1].

In contrast, RPNs whose role as a nurse was perceived as a drain on their individual resilience reported feeling like they could not afford to take a break because there was ‘not enough time’ [T45] and ‘just too much work (needed) to be done’ [T13]. In this way, their ‘dynamic role as a nurse’ drained their individual resilience because of their perception of their professional identity as a nurse to ‘keep going… no matter what’ [T68].

### 3.2 Preserving Self: ‘It's about compartmentalizing things’ vs. ‘You get consumed by [work]’

In the second theme, ‘Preserving Self’, RPNs spoke about the need to compartmentalize their work from other aspects of their life, rather than being entirely consumed by it. When the process of ‘Preserving Self’ was a drain on RPNs' resilience, participants felt that they were ‘consumed by’ [T17] their work. In contrast, when ‘Preserving Self’ was perceived as fuel for the process of maintaining individual resilience, participants could compartmentalize aspects of their work and ‘turn off [at] work’ [T18] as a coping strategy. This was manifested as a sense of preserving self, whereby coping through compartmentalization was an effective strategy to fuel individual resilience:

I've done a very good job of separating the two [work and home], and I think that, in order to be resilient, in order to be able to cope with the demands and the stress, you have to be able to not take it home with you [T81].

Along the same vein, other RPNs spoke about separating and detaching from work to maintain a work‐life balance. This was expressed to be fuel for their individual resilience whereby they were able to preserve themselves as separate entities from being an RPN by ‘turn[ing] it off’ [T1]:

I have to detach, like, as soon as I come home, I have to leave work at work, and I want to come home to a completely different environment … like a mini escape [T77].

RPNs described home as an ‘escape’ [T77] from their work. This speaks perhaps to a sense of entrapment in their role as an RPN driven by their sense of professional identity, responsibility and obligation as a nurse to keep going. When RPNs were able to compartmentalize to preserve their sense of self, this fueled their individual resilience:

At the end of my shift, I'm going to go home and I'm going to not be stressed about anything that happened here [at work; T34].

An aspect of being able to preserve the self, and thus maintain their individual resilience, was compartmentalization of work from home:

Tomorrow is a new day and there's going to be other challenges tomorrow. So, you have to be able to let it go but you need to have closure. You need to deal with it in the appropriate manner, and then move on to tomorrow [T56].

Being able to ‘deal with it’ [T56] was a challenge for some RPNs who lacked the workplace and leadership supports necessary to share their thoughts, fears, conflicts and challenges in the workplace with colleagues and/or leaders. At work, some RPNs described trying to ‘turn off’ [T18] and build an ‘iron suit’ [T64] of armour around themselves, as strategies that would preserve their sense of self to fuel their resilience:

Trying to, like, sort of ‘turn off’ when I'm off work. Which can be difficult at times, especially during COVID [T18].

The idea that RPNs needed to ‘turn off’ [T18] at work to get through the day may be a major signal for LTC administrators of the need for additional workplace supports for RPNs. RPNs inherently view their work as a professional obligation and face unique challenges at work that require them to react differently than they would in other aspects of their lives:

Nurses are put into situations that normal individuals wouldn't come across on a daily basis—and so things might be said to us that we would normally take personally, and they hurt our feelings, or we just wouldn't know how to react, and so those things require a lot of resilience in the sense that you have to understand the situation. You have to know how to react to it. You have to have kind of an iron suit, an iron mental suit, where you understand that this is not directed to me, this is not a personal attack or anything like that. So, strength in the sense that you have to be able to understand what the boundaries are, where the line is crossed, [and] the different realms of being an individual, of being a nurse [T64].

When a nurse is fueled by the ability to cope with ‘situations that normal individuals wouldn't come across on a daily basis’ [T64], their individual resilience is maintained. But when an RPN is considering a career change and describing the need for counseling, their consumption of work drains their resilience:

You keep getting consumed. You get consumed in the activity, you get consumed … sometimes I get home and I overthink, and you know I'm always … worried [T17].

RPNs talked about how compartmentalization at home for coping was achieved by embracing other aspects of their identity, like family, self‐care and parenting. Many talked about avoiding discussion of the pandemic at home, ‘turn[ing] off the TV’ [T101] to avoid the news and trying ‘not think about what's going on at work’ [T8]. In contrast, being consumed by their work, even at home, left RPNs feeling as though their individual identity and resilience were being drained. For some RPNs, being truly resilient may have meant accepting the limitations of their job and personal self.

### 3.3 Banding Together: ‘We're all in it together’ vs. ‘Started turning on each other’

The third theme, ‘Banding Together’, contained both fueling and draining experiences derived from individuals who felt a sense of connection versus a sense of conflict with their colleagues. When RPNs felt they were not working cohesively, and/or found fault with each other in the workplace, this marked an example of a drain on RPNs resilience. In contrast, when RPNs noted camaraderie and/or empathy amongst their colleagues, this was interpreted as fuel for the process of maintaining individual resilience under the same theme. These positive acts provided recognition for the significance of their work and encouraged teamwork and support from colleagues. RPNs either voiced that they had a cohesive team and a sense of banding together with their colleagues in LTC, or that they felt the pandemic had created a division amongst their colleagues:

[The pandemic] sort of had a polarizing effect on our workforce [T13].

This quotation speaks to the way that the pandemic influenced how individual resilience was enhanced or diminished by several factors relating to the nursing vocation. When it came to ‘Banding Together’, participants' interviews underscored contrasting opinions with respect to the sense of ‘team.’ Some expressed that they were frustrated with their teams, whilst others noted that their team became like family, sharing in a common struggle with common goals. Not banding together, however, was a drain on RPN individual resilience:

People would just leave work for me to do… I don't think we really focused on how we could support each other unfortunately [T43].

With the sense of professional identity as a nurse to care for patients despite contextual factors, leaving extra work was a burden for nurses who were already working in severely understaffed environments. Several strains imposed by the onset of COVID‐19 were perceived as contributing to tensions amongst colleagues. For instance, in addition to an increased workload, RPNs noted that the pandemic created workplace divisions when it came to policies and procedures, use of PPE, physical distancing and masking:

I always felt like many of [my colleagues] were not taking it seriously … we were supposed to be socially distancing ourselves…[colleagues] were starting to get very lax [sic] on lunch breaks, and sitting right beside you, and then taking the mask off, and eating… they didn't even wash [their] hands [T37].

This description highlights how some RPNs did not feel that they agreed with what they were trying to achieve with their colleagues, and what actions they needed to take to achieve it. As a result of these tensions, RPNs expressed an increase in conflict amongst their colleagues:

Everybody's kind of just turning on each other a little bit [T44].

This division amongst team members contributed to additional stress and frustration in the workplace:

Now people are just standoffish, and you know it's a little bit more toxic … you have to be careful with who you talk to and try to keep your chin up [T17].

The idea of keeping ‘your chin up’ [T17] speaks to their professional identity as a nurse to keep going despite the conflict, and the drain that ‘turning on each other’ [T44] had on their individual resilience. When RPNs did not band together with their colleagues, the social, professional and personal experiences they had at work were drains on their resilience, such that they struggled to maintain their professional identity. Staffing shortages, workload increases, fatigue and stress are all likely factors that contributed to the development of a ‘toxic’ [T17] environment at work. Even friendships at work were impacted by this lack of cohesion in core values and its resulting drain on individual resilience:

A lot of [coworkers] friendships have actually been affected by [the impact of the pandemic in LTC] just because everybody's so stressed and everybody's over it [T14].

The drain on nurses' capacity to adapt to challenge meant that, for some RPNs, there was nothing left in the tank to fuel their relationships because ‘everybody's over it’ [T14]. This suggests that RPNs had reached the absolute limit of their energy and abilities in the workplace. Emotions were reported to be running at high levels, with negativity, anger and stress reported by many RPNs, as an additional drain on their ability to ‘band together’:

The negative employees you didn't get much support from because they were very angry just to start out with going into work, they were already angry walking into work [T56];

The idea of having ‘negative employees’ [T56] implies the presence of other positive ones. This further reinforces the polarity observed in the Ontario LTC RPN workforce during the pandemic. In the presence of staffing shortages, increased workload, the absence of time off and the perceived inability to take breaks at work, negativity spilled out.

…sometimes you take it out on your coworkers… That's usually unfortunately what happens when you try to hold stuff in too long [T14].

This idea of taking ‘it out’ [T14] on coworkers demonstrates that RPNs need supports to express themselves during times of crisis. RPNs that did stick by their colleagues reported that banding together was an essential piece of maintaining their individual resilience:

Teamwork is how you be resilient in this job [T101].

Indeed, RPNs spoke of teamwork as being the conduit that fueled their individual resilience and sense of professional identity (i.e. to persevere despite a global pandemic):

Without working… as a team, you're not going to get through this [pandemic; T27].

Banding together with colleagues as a team provided participants' with the sense of being collectively united, which may have countered perceptions of abandonment or disregard for their work under difficult conditions. RPNs who banded together with their colleagues talked about an increase in team cohesion, and the importance of everyone having a role to play and ‘pitching in’ that fueled their individual resilience:

You need a good team. If you don't have a team that you can rely on it will be too much stress on you [T32].

This example speaks to the belief that everyone has a role to play, which is essential in bolstering perseverance in the workforce. This contributes to feeling less like ‘a number’ [T65] and more like a fundamental part of something bigger than yourself. In fact, RPNs even recognized that they worked together and accomplished tasks they did not want to do because of the sense of the greater good that ultimately was driven by their sense of professional identity as a nurse:

Everybody helped everybody else… they help without being asked. Everybody pitched in, they did things you know they didn't want to do [T7].

Banding together as a team was an essential dimension that fueled individual resilience in RPNs working in LTC during COVID‐19. The common struggles they shared both inside and outside of work were crucial to the formation of a cohesive team:

Because we were all struggling, and sometimes we were the only people [that knew what it was like], like the only family I know… I think we all kind of held on to each other, to kind of stay afloat [T32].

Being a member of the team, ‘banding together’, was described to be a positive factor in overcoming obstacles associated with the pandemic. Sharing a common struggle brought some RPNs closer together. Some talked about how their families or friends could not understand their experience inside the LTC home, but their team of colleagues ‘got it’ and this validated their experiences, fueling their resilience to cope and adapt to the pandemic.

### 3.4 Sense of leadership support: ‘It's all on you’ vs. ‘Saw [management] more than ever’

The final theme, ‘Sense of Leadership Support’, was also regarded by RPN participants in distinct ways, either as a fuel or drain on their individual resilience. RPNs voiced that they felt either abandoned by or supported by, their leaders during the pandemic. Some RPNs expressed that they were operating ‘on their own’ and had to make decisions independently (i.e. ‘executive decisions’ [T85]), whilst other RPNs voiced that ‘management’ had increased their presence in the LTC home and were lending a great deal of support to the staff, despite the increased workload in administration. Insufficient leadership support was described as a drain on individual resilience and RPNs felt that their profession was undervalued and positioned as ‘less than’ compared with ‘higher‐level’ administrators. In contrast, when leadership support was present, administrators acknowledged the significance of RPNs' work and encouraged the nurses to persevere. When RPNs' sense of leadership support was a drain on their individual resilience, they reported that:

Everybody has their positions – and their own job – so it's kind of all on you, or in this case, me, to get the job done [T101].

Abandonment was also described as a drain on RPNs' professional identity, leaving them feeling like they were alone in upholding their position in the workforce:

You're supported, but you're not supported. You're not supported with the fact that the workload's higher, and all these duties are on you, and if you don't do them, being told ‘you gotta [sic] do this right now, this is your responsibility’ [T6].

Many RPNs reported having an increase in their workload over the course of the pandemic. Additional safety measures, COVID‐19 screenings and PPE requirements consumed the time of RPNs, who were working in already stretched staffing environments. In contrast, when RPNs felt supported by their administrative leadership, this fuelled their individual resilience:

Right from the beginning, [leadership] were fully transparent with information… they worked tirelessly. They made sure we had everything we needed… none of them, none of them, had holidays last year. They were there all the time. The executive director came in at night… I was totally impressed with how they handled the situation [T7].

The individual resilience of RPNs was fueled when they were able to see their organizational leaders work as hard as themselves. This perceived equality between leadership and staff was important in reinforcing RPN professional identities as nurses with an obligation to patient care, despite the other contextual challenges they faced during the COVID‐19 pandemic. But RPNs' sense of leadership support was also reflected by how RPNs reported they had to alter their expectations of their leadership during the pandemic to experience less drain on their own capacity to adapt:

I lowered my expectations. I had really high expectations of… the people in management roles [before the pandemic; T65].

Having higher expectations of leadership before the pandemic suggests that RPNs acknowledge the unprecedented struggle associated with the COVID‐19 pandemic, and the lack of preparedness for LTC RPNs and administrators during this time. In other instances, several participants reported feeling confident and motivated by the support of their leadership team:

[Management] help staff to feel confident, and to feel motivated and positive about their work and I think that does makes you feel better as a professional in terms of what you can provide to patients, and what care and support you can give to patients [T57].

Being valued at work by leadership was important to RPNs. Having the support of their leaders allowed these RPNs to continue to work and maintain their capacity to adapt as challenges arose. RPNs acknowledged that their sense of support was related to the perceived presence or visible absence of leadership. RPNs who felt they received little support from their leadership noted:

Management's upstairs and the people who are getting the brunt of it are your colleagues [T4].

When management was perceived to be at a distance from the daily struggles of the RPNs in LTC home areas, they felt like they were attending to the frontline of this pandemic alone. This sense of abandonment drained the RPNs' individual resilience and challenged their professional identities as nurses. These experiences notwithstanding, several other RPNs had a different response:

[Leadership] are curious as to how staff are coping on the floor, so you do see them more often on the floor. They are asking more questions, seeing if staff have any concerns, and if there's any changes that could be done [T10].

The physical presence of leadership with and amongst the RPNs in LTC was an important factor that contributed to them feeling valued in the workplace, which consequently, fueled their individual resilience. The impact of leadership presence was repeatedly reflected in reports of feeling valued, or alternately, devalued, in their professional roles. Some RPNs spoke about feeling unimportant to their leaders:

There has to be a focus by management… on not just making you feel like a number [T65].

This description of being ‘a number’ [T65] to management highlights the perceived lack of value that RPNs experienced. The perception of being valued as a number instead of a skilled contributing member of the nursing workforce impacted their sense of leadership support:

These organizations [LTC]… don't even value you [T18].

The perceived devaluation of the nurse's role drained their resilience and challenged their sense of professional identity. In juxtaposition, when RPNs felt valued and cared for by their leadership, this enhanced their sense of resilience:

When I was taking a couple days off for mental health days, I did get a phone call from my manager, not in terms of reprimanding me for taking this time, but to find out, you know, ‘what's going on, do you need any support? Are you okay?’. And I thought, that really impressed me, because in the past they've never really done that [T49].

The professional conduct on the part of leadership influenced RPNs' own resilience and sense of professional identity. This is also reflective of the high need for support in LTC during the pandemic and the awareness needed from leadership that their staff was suffering under the weight of constant change and staffing shortages. Available resources, like employee assistance programs (EAPs), were described by participants as unhelpful and RPNs spoke about needing access to greater support during the pandemic. Some RPNs expressed wishing they could have relied on their leadership teams during trying times:

It would have been nice if we could actually rely on our upper management for support. There were a lot of times when we would tell them what was going on and they would always say, ‘get the job done’ [T32].

RPNs wanted to be acknowledged as more than just ‘a number’ [T65]. They wanted to feel secure expressing their own struggles during this challenging time, with fear of reprisal. The idea that RPNs were told to ‘get the job done’ [T32] regardless of other factors (e.g. staffing shortages, death, conflict) indicates that some leadership teams lacked the awareness or capacity to adequately support their team members. The significance of having support through stressful times was emphasized by participants identifying a desire for an outlet to express their concerns. Many RPNs voiced that having someone to talk to in LTC during the pandemic would have been helpful:

It would be nice to have [support in the moment], and I know we're supposed to rely on our managers, upper management, and right now at the moment [sic] they're not focused on that… so they would always just turn around and say ‘well call EAP [Employee Assistance Program]’… in the moment it's not very helpful. It would be helpful later, to maybe have a counsellor or two on site [T32].

This idea of having support available in the workplace was peppered throughout the nurses' interviews. Some management and leadership teams were unable to provide the mental and physical support RPNs needed to sustain their individual resilience over the grueling pandemic.

## 4 DISCUSSION

This study presents a unique emergent conceptual model to describe how RPNs maintain individual resilience during a crisis like COVID‐19. The process includes the following four themes: ‘Dynamic Role of the Nurse’ and ‘Preserving Self’, ‘Banding Together’ and ‘Sense of Leadership Support’. These themes were found to either encourage or deplete individual resilience, in unique combinations, in a process whereby RPNs fundamentally drew on their professional identity as a nurse. To our knowledge, this is the first study to examine resilience in RPNs working in LTC during COVID‐19. Resilience is important in nurses' personal and professional lives because it helps to protect them from the negative consequences of workplace stressors. Strategies, resources and workplace support that can promote self‐care and resilience could help rejuvenate and retain the Ontario RPN workforce in their dynamic role.

Our findings echo previous research demonstrating that during COVID‐19, many healthcare workers (i.e. doctors, nurses, other interdisciplinary staff and office workers) experienced psychiatric symptoms, suggesting that better support and interventions to protect the mental health of these workers are vital (Awano et al., 2020). Our study affirmed that a sense of leadership support is an important factor in maintaining individual resilience and that participants required additional mental health supports: something that has been considered in this workforce long before COVID‐19 (Woodhead, Northrop & Edelstein, 2016). Previous research demonstrates that job demands (e.g. greater occupational stress) are associated with more emotional exhaustion, more depersonalization and less personal accomplishment (Woodhead, Northrop & Edelstein, 2016). Although the current study did not directly measure mental health, findings from Japan suggest that 10% of healthcare workers developed moderate‐to‐severe anxiety disorders, and roughly 28% developed depression during COVID‐19 (Awano et al., 2020). Problems with anxiety and fear of infection and death, isolation and unreasonable treatment and motivation and escape from work were higher in the group who experienced depression relative to the group who did not. This is also demonstrated by research studying front‐line nursing home staff experiences during COVID‐19 in Ontario (White et al., 2021). Our participants reported a need to preserve their sense of self, commonly through the strategy of compartmentalization. As research suggests that older healthcare workers, and healthcare workers with higher resilience, may be less likely to develop depression (Awano et al., 2020), the importance of resilience for the health of our healthcare workforce is clear.

Researchers in Indonesia found a significant correlation (i.e. Spearman's correlation = −0.519) between the level of resilience, as measured by the Connor–Davidson Resilience Scale (CR‐RISC), and anxiety, as measured by the State–Trait Anxiety Inventory (STAI) questionnaire, in hospital healthcare workers during COVID‐19 (Setiawati et al., 2021). The authors concluded that there is a negative correlation between levels of resilience and the degree of anxiety experienced. Our participants described that they felt supported when leadership was visible and highlighted their specific need to have a counselor on site available for staff as required. These findings align with the currently submitted work from the present research team (Connelly et al., 2022), which surveyed 381 RPNs working in Ontario LTC homes. Survey respondents reported extreme levels of job (54.5%) and personal (37.8%) stress and reduced physical and mental health during the pandemic when compared with their retrospective self‐reported stress levels before the pandemic. The erosion of resilience secondary to the impacts of COVID‐19 was evident for RPNs working in LTC. Overall resilience, as measured by the Resilience at Work Individual and Team Scales, was low compared with other samples in the literature. Study respondents scored the poorest on the subscales of measuring things like stress and self‐care. These findings are mirrored by our theme of ‘preserving self’ where some RPNs reported being consumed by their work and failing at work‐life balance.

Like our findings, research conducted in British Columbia, Canada with nurses in LTC homes also identified room for improvement in communication and staffing practices and policies (Havaei et al., 2021). Our participants perceived their leaders as providing support when they stayed at work and missed their holidays. This might suggest that to fuel RPN's resilience, the perception of leaders going above and beyond their regular call of duty may be advantageous.

To address the systemic problems of staffing shortages and burnout in LTC that have been amplified by COVID‐19, we must consider the change to systems, policies and practices in the short‐ and long‐term. Healthcare or organizational resilience is defined as the capacity to adapt to challenges and changes at different systems levels, to maintain high‐quality care. ‘Capacity’ refers to a ‘set of capacities’ (e.g. the materials and resources that underpin resilience) at individual, team and system levels (Wiig et al., 2020, p. 6). ‘Adapt’ refers to a process of how nurses ‘continue to operate and deliver services despite stress, disruptions, unforeseen events, and insufficient resources and competence’ (Wiig et al., 2020). Fused together, capacity and adaptation signal the need for increased resources that might bolster resilience. Nurses in this study talked about their dynamic role, and their need to make decisions independently despite inadequate workplace processes to help them to do so (i.e. a gap in the organizational resilience of LTC homes). In the LTC sector, supports must be in place to fuel individual resilience to allow nurses to effectively enact their role.

Researchers from the United States suggest strategies for changing systems, policies and procedures such as requiring uniform public reporting of COVID‐19 cases in all LTC settings; identifying and supporting unpaid caregivers; bolstering protections for the direct care workforce; increasing coordination between public health departments and LTC agencies and providers; enhancing collaboration and communication across health, LTC and public health systems; further reducing barriers to telehealth in LTC; providing incentives to care for vulnerable populations (Dawson et al., 2021). The focus should be on workforce development, funding reform and the creation of a sustained age‐friendly health system, inclusive of a resilient healthcare workforce.

For the profession of RPNs, leadership support needs to increase, and opportunities need to be provided for RPNs to work as a team. Currently, in the RPN profession, there is a juxtaposition between the nursing role and the professional obligations of the job. As a result of poor work‐life balance, being consumed by work, and being exposed to trauma, RPNs may require greater access to sick leave provisions and mental health resources. Additionally, findings from this study suggest a needed emphasis on the immediacy of support at the moment. Additionally, long‐term support is needed to address vicarious trauma and secondary traumatic stress in healthcare professionals (Guitar & Molinaro, 2017).

### 4.1 Limitations

To our knowledge, this is the first study describing the process of how RPNs maintain individual resilience whilst working in LTC homes during the COVID‐19 pandemic. This is a qualitative constructed grounded theory study; therefore, the generalizability of these findings is limited. The study was conducted only in the province of Ontario, as a result, data may have been different if collected across other provinces. In addition, there is the opportunity of social desirability bias influencing the responses of participants in our interviews. Our interview data also only collected the experiences of those who were willing to participate and share their experience, which could skew the results to reflect this sub‐group of RPNs. RPNs with ‘less’ resilience may not have responded to the survey at a time of extreme workload pressures and general stress. In this study, information about how many RPNs had left their employment in LTC homes in Ontario was not collected.

There are also some methodological limitations associated with the use of the Zoom platform for data collection. Technological competence may be challenged by audio and visual impairments, or other physical limitations which may make it difficult to use the computer, tablet, or phone (Howlett, 2021). Video conferencing can be unreliable when the internet is unstable, resulting in problems (audio cut‐offs) that would not happen in person. Howlett (2021) suggests that online research is now an equally valid approach to research as in‐person interactions and noted that her participants were noticeably more comfortable engaging in research online than in in‐person interactions.

## 5 CONCLUSION

Resilience in RPN study participants working in LTC during the pandemic was described as largely individual resilience. Study findings informed two distinct perspectives on nurses' resilience in LTC, one where nurses were able to maintain resilience (i.e. sustaining) and another where they were not (i.e. fraying). Sustaining and fraying resilience presented as bimodal processes; four themes were identified, each constructed of both sustaining and fraying experiences: ‘Dynamic Role of the Nurse’; ‘Preserving Self’; ‘Banding Together’; ‘Sense of Leadership Support’. Resources to support self‐care and work‐life balance are needed, as are workplace supports to build capacity for team‐based care practice(s), collegial supports in problem‐solving and opportunities for ‘connecting’ with ‘LTC nursing colleagues’. Understanding RPNs' experiences during the COVID‐19 crisis is critical to inform the development of social and institutional policy and retention and recruitment strategies. Current policies, whilst designed to promote better outcomes for people living in LTC and their care partners, may not be sufficient to prepare RPNs and the LTC workforce with the capacity to adapt to stressors and unexpected events or crises important for maintaining a culture of care. The likelihood of future strain arising from changing societal expectations, increased demand for services and the need for specialized geriatric knowledge with the expanding aging demographic (Ontario Health Coalition, 2019), necessitates the development of evidence‐informed strategies to address these increasing demands. In future research, it would be beneficial to align the work responsibilities with demonstrated professional competencies and skills of the workforce. Findings suggest a role for professional development and workplace solutions for rebuilding this critical workforce to continue caring for older adults living in LTC as vulnerable members of our society. Resilience is important for the retention and recruitment of nurses in LTC homes and future studies need to examine this construct in relation to objectively measured staff and resident outcomes.

## AUTHOR CONTRIBUTIONS

Denise Connelly—Principal investigator, obtained grant funding, contributed to the design of the work, revised the article and approved the version to be published. Anna Garnett—Contributed to the design of the work, revised the article and approved the version to be published.Nancy Snobelen—Contributed to the design of the work, revised the article and approved the version to be published. Nicole Guitar—Contributed to the design of the work, conducted data analysis and interpretation, drafted the article, revised the article and approved the version to be published. Cecilia Flores‐Sandoval—Contributed to the design of the work, performed data collection, assisted with data analysis and interpretation and drafting of the article. Samir Sinha—contributed to the design of the work, revised the article and approved the version to be published. Jen Calver—Contributed to the design of the work, revised the article and approved the version to be published. Diana Pearson—Contributed to the design of the work, revised the article and approved the version to be published. Tracy Smith‐Carrier—Contributed to the design of the work, revised the article and approved the version to be published.

## FUNDING INFORMATION

This research was funded by a Social Sciences and Humanities Partnership Engagement Grant in partnership with the Registered Practical Nurses Association of Ontario (WeRPN).

## CONFLICT OF INTEREST

No conflict of interest has been declared by the author(s).

### PEER REVIEW

The peer review history for this article is available at https://publons.com/publon/10.1111/jan.15453.

## Supporting information


Appendix S1

## Data Availability

The authors does wish to share the Research data.
